# Knowledge, Attitudes, and Practices Toward Blood Pressure Control Among Refugees Resettled in the United States

**DOI:** 10.1007/s44197-025-00487-7

**Published:** 2025-11-28

**Authors:** Abeer Alharthi, Oluwabunmi Ogungbe, Yvonne Commodore-Mensah, Tala Al-Rousan

**Affiliations:** 1 King Saud University College of Nursing, Riyadh, Saudi Arabia; 2 Johns Hopkins University School of Nursing, Baltimore, MD USA; 3 Johns Hopkins University Bloomberg School of Public Health, Baltimore, MD USA; 4 Herbert Wertheim School of Public Health and Human Longevity Science, University of California, San Diego, CA USA

**Keywords:** Blood pressure, Iraqi, Syrian, Refugees, KAP, Hypertension, Refugee health, Cardiovascular health

## Abstract

**Background:**

Forced displacement has created widespread health disparities, with refugees experiencing disproportionate risks of chronic conditions like hypertension. In the United States, a limited understanding of how knowledge, attitudes, and practices (KAP) shape blood pressure (BP) outcomes continues to hinder equitable care. This study explores the relationship between KAP and BP control among Iraqi and Syrian refugees who resettled in the United States.

**Methods:**

Iraqi and Syrian refugees (*n* = 95) with hypertension at a federally qualified health center in San Diego, California, participated in this study. They completed a survey in Arabic assessing KAP based on a scale validated in non-English speakers and were instructed to do home BP monitoring using digital cuffs for at least 3 days per week for 4 weeks. The outcome was BP control based on the American College of Cardiology (ACC) criteria, calculated from an average of home BP readings. Mixed-effect logistic regression was used to assess the relationship between each KAP quartile and BP control. KAP quartile was categorized into (poor, fair, good, excellent).

**Results:**

Participants were 56% male, and the mean age was 58.8 (± 15.97) years. 86% were unemployed, 40% had at least a bachelor’s degree, 63% had limited English proficiency, and only 3.6% had an annual income of ≥$35,000. Mean BP was 129/79 mmHg (SD systolic: 22.17 mmHg; SD diastolic: 11.15 mmHg). Scores were computed for each KAP sub-scale: knowledge (0–16), attitude (1–20), and practice (1–8). Knowledge 13.2 (± 2.37), attitude 14.6 (± 2.38), and practice 6.5 (± 1.17). Higher knowledge levels were associated with higher odds of BP control (OR 1.89, 95% CI 1.39–2.55). An inverse relationship was observed between attitudes and BP control: participants with “Fair” and “Good” scores had reduced odds of BP control (OR = 0.63, 95% CI: 0.52–0.77; and OR = 0.58, 95% CI: 0.45–0.75, respectively).

**Conclusions:**

These findings highlight the urgent need for culturally grounded health education and systems-level strategies that address both informational and perceptual barriers to BP control. Advancing health equity for refugee populations demands interventions that go beyond awareness, integrating trust-building, accessibility, and empowerment into chronic disease management.

**Supplementary Information:**

The online version contains supplementary material available at 10.1007/s44197-025-00487-7.

## Background

 As of June 2024, the United Nations High Commissioner for Refugees (UNHCR) reported that 1.0% of the global population is displaced due to war and conflict, representing the largest surge in forced displacement ever recorded [1]. The number of forcibly displaced individuals is projected to reach 1.2 billion by 2050 due to increased economic inequality, natural disasters, and political unrest [2]. A substantial portion of the global refugee population originates from a few countries, notably Syria and Iraq, which together have produced over 8 million refugees [3, 4]. The United States (US) has historically resettled more refugees than any other country [5]. This growing demographic increasingly intersects with public health challenges [2]. Among these is hypertension, a critical issue in the US, affecting nearly half of the adult population and significantly contributing to morbidity and mortality [6, 7].

The complications associated with uncontrolled Blood Pressure (BP), such as heart disease, stroke, and kidney failure, impose substantial costs on the healthcare system, straining resources and increasing the economic burden of chronic disease management, which reached $198 billion yearly [8]. Additionally, a significant health disparity persists among racial and ethnic minority groups [9]. The Surgeon General’s Call to Action on Hypertension further emphasizes the need to address hypertension in minority populations as a national priority [10]. These health disparities are influenced by a range of factors, including migration, psychosocial stressors, socioeconomic status, access to healthcare, and cultural beliefs about health and illness [10–12].

Refugees who were displaced from the Middle East and North African (MENA) region, a major producer of refugees globally, and resettled in the U.S. face unique challenges that contribute to higher rates of uncontrolled BP [9, 12, 13]. There is mounting evidence reporting social disadvantages in this population, such as living in poverty, language barriers, dealing with mental health trauma, and experiencing unstable housing and discrimination [14, 15]. In addition, the stress of displacement and the challenges of accessing culturally concordant healthcare in host countries exacerbate the risk of chronic health conditions [12, 16]. Despite research investigating the overall negative social determinants of health, factors affecting hypertension control among refugees remain understudied [17].

Previous research has indicated that one crucial prevention strategy for controlling BP is harnessing knowledge, attitudes, and practices (KAP) [18, 19]. KAP surveys have been utilized in prior research to understand population behavior and healthcare-seeking practices toward hypertension management [20]. However, there remains a gap in their application, specifically among Arab refugees. This paper is among the first to investigate the association between KAP and BP control among Iraqi and Syrian refugees resettled in the U.S.

## Methods

### Data Source

This study is part of a larger pilot investigation of hypertension self-management behaviors among Arab refugees residing in San Diego, California [21]. San Diego is a major refugee resettlement hub and hosts the majority of the Arab refugee population resettled in the U.S., besides other refugees from all over the world [8, 22, 23]. Participants were trained on how to use connected BP cuffs and measure their BP at home and interviewed using semi-structured interviews. They were recruited through a federally qualified health center using a patient list of patients in a refugee-majority neighborhood with a documented diagnosis of hypertension. An Arabic-speaking research assistant called participants to ask about their interest in participating in this study. Almost 90.0% of those contacted indicated interest in participation. Participants were included if they met the inclusion criteria and were able to provide informed consent. All participants participated in an educational session on BP device utilization and measurements. Research demonstrated BP monitoring, and the participant was included if they were able to complete a mock session on the use of the BP monitor, measure BP, and record measurements per protocol. The baseline interview included a KAP questionnaire. All participants were asked to measure their BP at home using connected BP cuffs at least 3 times/week for 4 consecutive weeks. BP measurements were transferred in real-time to a software that records BP readings and is only accessible to the study team.

## Participants

The study sample included (*n* = 109) Syrian and Iraqi refugee participants who were previously diagnosed with hypertension and were recruited into this study between October 2021 and May 2022, using a pre-screened patient list from the El Cajon (refugee-majority neighborhood) refugee clinic of the Family Health Center of San Diego (FHCSD), a federally qualified health center.

Inclusion criteria were being 18 years of age or older; having a physician-confirmed diagnosis of hypertension documented in the electronic health record; verified resettlement in the United States under refugee status; and being originally from Iraq or Syria. Participants were excluded if they were currently pregnant, of childbearing age without contraceptive use, or had a history of end-stage renal disease, recent cardiovascular events or hospitalization for unstable angina within the past three months, symptomatic heart failure within the past six months, or a left ventricular ejection fraction below 35% .

## Measure

We conducted a literature review of all KAP measures and concluded that none were validated in Arabic speakers. We thus adapted a modified version from a previously developed KAP tool and examination of BP validated in other languages, which includes a structured questionnaire that captures data on what participants know about a health condition (knowledge), their beliefs concerning the condition’s severity (attitude) and their susceptibility to it, and their behaviors or practices related to managing or preventing the condition (practice) (Table 1) [24]. Cronbach’s alpha was used to assess the internal consistency and reliability of the KAP subscales (See Additional File 1).

**Table 1:** Knowledge, Attitude, and practice (KAP) on hypertension among refugees

KAP items	Answer
Knowledge	
Hypertension causes cardiovascular disease	Yes No I do not know
Hypertension causes brain damage	Yes No I do not know
Diet rich in salt causes high BP	Yes No I do not know
Being overweight causes high BP	Yes No I do not know
Smoking causes high BP	Yes No I do not know
People with high BP show symptoms	Yes No I do not know
People with high BP should take medications for many years	Yes No I do not know
Can give a value when asked about own BP	Yes No I do not know
**Attitude/Perception**	
Has a doctor ever told you that you have hypertension	Yes No I do not know
Thinks hypertension is controlled	Yes No I do not know
Receives treatment for hypertension	Yes No I do not know
Rates their overall health	Good, excellent Fair Poor
Considers to be less healthy than a person of the same age who has been living the majority of their life in the United States	Yes No I do not know
Thinks they could get an MI or stroke in next year	Always Sometimes Never
Thinks can do things to prevent themselves from getting a heart attack or stroke that affects brain	A little more A few things Nothing
Thinks can do much to improve own health	A lot more A little more Not much
Thinks lifestyle can greatly influence health in the future	A lot more A little more Not much
**Practice**	
Visited a doctor in the last 12 months	More than 4 times 2–4 times 0-1times
Last time had BP checked	Less than 2 months ago 6–12 months ago more than a year
Took non-traditional medicine for any reason during last 4 weeks	Yes No I do not know
Smoke cigarettes	Yes No
MI indicates myocardial infarction	

### Statistical Analysis

To facilitate a consistent analytic approach, the response options for the KAP tool were calibrated into a binary and hierarchical format. For binary items, responses were designated as ‘yes’ (score of 2) or ‘no’ (score of 1), with ‘I do not know’ capturing the absence of knowledge (score of 0). For items evaluating the extent of a response, a graded scale was applied: ‘a lot more’ (score of 2), ‘a little more’ (score of 1), and ‘not much’ (score of 0). This restructuring allowed for a unified response metric across diverse question types. Subsequently, scores were tallied to form sub-scale scores within the domains of Knowledge (0–16), Attitude (1–20), and Practice (1–8), with higher aggregates reflecting more positive attributes in each respective domain. Scores were categorized into quartiles (poor-excellent). The outcome was BP control, calculated from an average of self-measured BP readings over 3 days for 4 weeks.

Descriptive statistics were used to summarize the demographic and clinical characteristics of the study participants. Continuous variables were reported as means ± standard deviations (SD) or medians with interquartile ranges (IQR), while categorical variables were reported as frequencies and percentages. Mixed-effects logistic regression models were used to examine the associations between the KAP measures and BP control, defined as systolic BP < 130 mmHg and diastolic BP < 80 mmHg per the ACC criteria [25]. Unadjusted and adjusted odds ratios (ORs) with 95% confidence intervals (CIs) were reported [25]. The adjusted models controlled for sex, country of origin, length of stay, and insurance status. All statistical analyses were conducted using Stata/SE (version 18.0). A two-sided p-value < 0.05 was considered statistically significant.

## Results

### Sample Characteristics

The final analysis included 95 refugees from Syria and Iraq. Fourteen were excluded because they had not been using the BP monitor and had missing BP data. Among them, 49.5% were male, with an average age of 59 years; 81.1% were from Iraq, and 18.9% from Syria. A majority, 83.2%, were married, while 86.3% were not employed. Almost 99.0% were insured, and 40.0% had attained at least a bachelor’s degree. In addition, 63.2% did not speak or write English proficiently. (Table 2.).

**Table 2.** Controlled Hypertension per ACC Criteria for Refugees Diagnosed with Hypertension and Receiving Treatment

	**Not Controlled hypertension**	**Controlled hypertension**	**Total**	**p-value**
**N**	58 (61.1%)	37 (38.9%)	95 (100.0%)	
Age (Mean ± SD)	59.255 (11.943)	58.121 (20.959)	58.813 (15.970)	0.738
**Sex**				
Female	35 (60.3%)	13 (35.1%)	48 (50.5%)	0.017*
Male	23 (39.7%)	24 (64.9%)	47 (49.5%)	
**Country of Origin**				
Iraq	45 (77.6%)	32 (86.5%)	77 (81.1%)	0.280
Syria	13 (22.4%)	5 (13.5%)	18 (18.9%)	
**Marital Status**				
Divorced	3 (5.2%)	0 (0.0%)	3 (3.2%)	0.395
Married	46 (79.3%)	33 (89.2%)	79 (83.2%)	
Never married	1 (1.7%)	0 (0.0%)	1 (1.1%)	
Widowed	8 (13.8%)	4 (10.8%)	12 (12.6%)	
**Employment**				
No	52 (89.7%)	30 (81.1%)	82 (86.3%)	0.236
Yes	6 (10.3%)	7 (18.9%)	13 (13.7%)	
**Children**				
No	2 (3.4%)	0 (0.0%)	2 (2.1%)	0.254
Yes	56 (96.6%)	37 (100.0%)	93 (97.9%)	
**Education**				
Bachelor’s degree	24 (41.4%)	14 (37.8%)	38 (40.0%)	0.328
High school	6 (10.3%)	9 (24.3%)	15 (15.8%)	
Less than high school	15 (25.9%)	5 (13.5%)	20 (21.1%)	
Postgraduate degree	11 (19.0%)	8 (21.6%)	19 (20.0%)	
Vocational certificate	2 (3.4%)	1 (2.7%)	3 (3.2%)	
**English language Proficiency**				
No	38 (65.5%)	22 (59.5%)	60 (63.2%)	0.551
Yes	20 (34.5%)	15 (40.5%)	35 (36.8%)	
**Medical Insurance**				
Don’t’ know	0 (0.0%)	1 (2.7%)	1 (1.1%)	0.208
Yes	58 (100.0%)	36 (97.3%)	94 (98.9%)	
**Smoking**				
Cigarettes, Hookah/Shisha/Argeelah	0 (0.0%)	2 (5.4%)	2 (2.1%)	0.225
Hookah/Shisha	1 (1.7%)	0 (0.0%)	1 (1.1%)	
Cigarettes	9 (15.5%)	9 (24.3%)	18 (18.9%)	
Hookah/Shisha/Argeelah	4 (6.9%)	1 (2.7%)	5 (5.3%)	
Not smoking	44 (75.9%)	25 (67.6%)	69 (72.6%)	
**Years since resettlement in US**				
Years (mean ± SD)	12.017 (3.601)	11.806 (4.104)	11.936 (3.781)	0.793
**Blood Pressure**				
Systolic (mean ± SD)	140.603 (19.430)	111.622 (12.608)	129.316 (22.173)	< 0.001*
Diastolic (mean ± SD)	85.603 (7.843)	68.703 (6.948)	79.021 (11.155)	< 0.001*

*Indicates significant value

## Sociodemographic Characteristics by Blood Pressure Control Status among Refugees Diagnosed with Hypertension and Receiving Treatment

Males were more likely to have controlled hypertension than females (*p* = 0.017). No significant differences in BP control were observed by country of origin, marital status, employment, or English language proficiency (Table 2.). In contrast, BP measurements themselves differed significantly between the two groups, with those in the controlled category showing substantially lower systolic and diastolic pressures (*p* < 0.001 for both) (Table 2.).

## Knowledge, Attitudes, and Practices on Hypertension

Table 3 presents the association between KAP scores and BP control. This analysis was conducted using both unadjusted and adjusted logistic regression models, with the latter controlling for age, sex, country of origin, duration of residence in the U.S., and health insurance status. Participants with “excellent” knowledge had higher odds of achieving BP control compared with those with “poor” knowledge (OR = 1.89, 95% CI 1.39–2.55). In contrast, “fair” and “good” attitude scores were associated with lower odds of control (OR = 0.63, 95% CI 0.52–0.77 and OR = 0.58, 95% CI 0.45–0.75, respectively), while “excellent” attitudes were associated with better control (OR = 1.27, 95% CI 1.03–1.56). Practice scores did not show a significant association with BP control.

**Table 3:** Knowledge, Attitude, and practices and blood pressure control among Iraqi and Syrian refugees who settled in the U.S. *N* = 95

	Unadjusted	Adjusted**
Knowledge		
Poor	**1.00**	**1.00**
Fair	1.17 (0.96–1.44)	1.20 (0.98–1.48)
Good	1.32 (1.08–1.62)*	1.30 (1.06–1.59)*
Excellent	2.15 (1.60–2.88)*	1.89 (1.39–2.55)*
Attitude/Perception		
Poor	**1.00**	**1.00**
Fair	0.62 (0.51–0.75)*	0.63 (0.52–0.77)*
Good	0.56 (0.44–0.72)*	0.58 (0.45–0.75)*
Excellent	1.29 (1.05–1.58)*	1.27 (1.03–1.56)*
Practice ***		
Poor	**1.00**	**1.00**
Fair	0.86 (0.73–1.02)	0.90 (0.75–1.08)
Good/Excellent	0.92 (0.76–1.12)	1.01 (0.81–1.24)

* Values significant at *p* < 0.05.

** Adjusted for age, sex, country of origin, length of stay, & health insurance

*** Scores were divided into tertile because no participants scored in quartile 3 when scores were divided into quartiles

## Discussion

Our findings highlight several significant results regarding hypertension control and associated factors among Syrian and Iraqi refugees resettled in the U.S. Sex emerged as a key factor, with males more likely to achieve BP control than females. Several studies have demonstrated that females are less likely to receive guideline-recommended antihypertensive therapy and are consequently less likely to achieve optimal BP control compared with males [26]. In contrast, other research has shown that although females may be more likely to receive treatment, they still experience poorer BP control outcomes. For example, a study among African immigrants found that although females were more likely than males to report taking antihypertensive medication, males were more likely to have their BP controlled [27]. This may suggest that achieving optimal BP control depends not only on treatment access but also on adherence and physiological and psychological factors. Additionally, refugee females in particular face compounded barriers, including caregiving roles, language limitations, and health-related anxiety that can interfere with consistent monitoring and treatment adherence [21].

BP measurements showed that participants with controlled hypertension had better systolic and diastolic readings compared to those with uncontrolled hypertension. Additionally, knowledge was positively associated with BP control. In contrast, participants with “Fair” or “Good” attitudes had lower odds of achieving BP control compared to those with “Poor” attitudes. Although participants with “Excellent” attitudes were more likely to have controlled BP.

In our analysis, we identified that knowledge about hypertension plays a crucial role in its management among Arab refugees resettled in San Diego. The findings are consistent with previous research that demonstrated good knowledge of hypertension is associated with improved medication adherence, increased BP control, and overall reduced morbidity and mortality [19]. In addition, a qualitative study showed that insufficient knowledge of hypertension symptoms was a significant barrier to medication adherence among Middle Eastern refugees [28]. Similarly, a study assessing hypertension KAP among hypertensive patients in Lebanon reported that patients had limited knowledge about hypertension, with misconceptions about traditional treatments persisting [19].

Interestingly, our analysis yielded that a fair attitude/perception correlates with lower BP control compared to poor, while an excellent attitude scored higher than poor. This outcome might indicate that attitudes towards hypertension vary and are influenced by factors such as geographical location, education level, length of time since hypertension diagnosis, and smoking habits [19]. Factors such as perceptions of illness and trust in healthcare providers may also impact hypertension management. In the Australian study, Arab refugees and migrants highlighted their distrust of healthcare professionals and the overmedicalization of healthcare in the host country, which negatively influenced treatment adherence [28].

BP practices were not associated with better BP control, suggesting potential inconsistencies within the unique demographic characteristics of our study population. This gap may stem from a range of beliefs and behaviors, including forgetfulness in taking medications, doubts about their necessity or effectiveness, and, in some cases, concerns about possible adverse effects [28]. Additionally, research suggests that refugees often lack access to home BP monitors and instead rely on public devices available at local pharmacies for measurement [21]. While some refugees found this approach helpful, others described significant challenges associated with using public BP devices, including long wait times that contributed to stress and concerns about privacy and social discomfort [21]. Furthermore, economic hardships further exacerbate these issues. Extreme poverty limits refugees’ ability to make healthy lifestyle changes, afford antihypertensive medications, or even travel to clinics for care [29, 30].

Our analysis of sociodemographic status and BP control showed that systolic and diastolic BP readings are significantly lower in the controlled group, which is expected. These results suggest BP control is achievable among refugees receiving treatment, regardless of demographic differences. Overall, the differences in KAP levels between the participants emphasize the unique health needs of this US-resettled refugee population. Despite the high burden of hypertension in this population, there remains a striking lack of empirical research examining KAP and their associations with BP outcomes in refugee groups. This gap limits the understanding of the mechanisms that influence disease control and impedes the development of culturally responsive interventions.

### Limitations

To our knowledge, our study is the first to examine the association between KAP and BP control in Syrian and Iraqi refugees resettled in the U.S. Our findings can be used to understand the behaviors of hypertensive refugees to better assist with self and home management practices.

It is important to note that our study had some limitations. Due to the nature of the tool used, other specific information may not have been captured. As a result, key factors that could significantly affect hypertension control, such as cultural beliefs and access to healthcare, might not have been included. Selection bias may also exist, as participants who were excluded had not been using their BP monitors and had missing BP data. The sociodemographic characteristics of the study sample may not be representative of broader refugee populations resettled in the U.S. For example, refugee populations typically have low participation in higher education, with global tertiary enrollment estimated at only 9.0% [31]. Additionally, most study participants were insured, which contrasts with the typical experience of refugees resettled in the U.S, who often face limited access to health insurance coverage [32]. Furthermore, some subscales demonstrated lower internal consistency, likely due to the multidimensional nature of attitudes and behaviors, which capture diverse components of hypertension management rather than a single homogeneous construct [33].

Answers to the survey could have been subject to recall bias. This study is a cross-sectional analysis that captured associations between KAP at the start of the intervention and BP measurements and didn’t account for changes over time or imply causation.

Overall, while this research does not aim to represent all refugees as this population is diverse and representative of many ethnic backgrounds, it seeks to provide insights into the KAP related to BP management among Syrian and Iraqi refugees and nuanced hypothesis generation related to the experience of displacement, which highlights areas for further investigation. Future longitudinal and qualitative analyses are needed to determine the role of acculturation and clinical and public health intervention targets.

## Conclusions

This study examined the role of KAP on BP control among Syrian and Iraqi refugees. The findings highlight the vital role of knowledge in hypertension management behaviors, which is essential in preventing CVD morbidity and mortality. While attitudes and practices showed complex influences on BP control, these findings advocate for further investigation of knowledge, attitudes and practices for managing hypertension among refugees and factors influencing them. Culturally sensitive public health interventions to improve KAP and inform future research on refugee health behaviors and long-term hypertension management are needed to improve BP control among refugees in the pursuit of optimal cardiovascular health for all.

## Supplementary Information

Below is the link to the electronic supplementary material.

Supplementary Material 1(DOCX 14.6 KB)

## Data Availability

No datasets were generated or analysed during the current study.

## Abbreviations

BP: Blood Pressure
CVD: Cardiovascular Disease
KAP: Knowledge, attitude, and practice
