# Supplementary material for: Knowledge, Attitudes, and Practices Toward Blood Pressure Control Among Refugees Resettled in the United States
Source: J Epidemiol Glob Health. 2025 Nov 28;15(1):141. doi: 10.1007/s44197-025-00487-7 (PMC12662913; doi:10.1007/s44197-025-00487-7)
Supplement: Supplementary file 1 — Supplementary Material 1(DOCX 14.6 KB) [file 44197_2025_487_MOESM1_ESM.docx]

| **Table 1: Cronbach's Alpha of the Knowledge, Attitude, and Practice (KAP) Scale** | | |
| --- | --- | --- |
| **Subscale** | **Number of Items** | **Cronbach's Alpha (α)** |
| **Knowledge** | 8 | 0.662 |
| **Attitude and Perception** | 9 | 0.348 |
| **Behavior** | 4 | 0.153 |
